# Potential challenges of implementing the Community Health Extension Worker programme in Uganda

**DOI:** 10.1136/bmjgh-2018-000960

**Published:** 2018-08-10

**Authors:** James O’Donovan, Christina Elise Stiles, Deogratias Sekimpi, Isaac Ddumba, Niall Winters, Edward O’Neil Jr

**Affiliations:** 1 Department of Education, University of Oxford, Oxford, UK; 2 Department of Research, Omni Med Uganda, Mukono, Uganda; 3 Uganda National Association of Community and Occupational Health, Kampala, Uganda; 4 Department of Community Health, District Health Office, Mukono, Uganda; 5 Department of Research, African Research Centre on Aging and Dementia, Mukono, Uganda

**Keywords:** health services research, health systems, public health

**Summary box**

- The proposed roll out of the Community Health Extension Worker (CHEW) programme is due to take place in Uganda in 2018 at an estimated cost of US$102 million over a 5-year period.
- Although this is a welcome move towards supporting the existing Village Health Team (VHTs) cadre of community health workers, several challenges and potential solutions are raised in this article.
- Uncertainties remain around potential tensions that may arise between current VHTs and the new CHEWs, the logistical implementation of the programme and financial sustainability.
- Prior to roll out of the CHEW programme, greater attention must be given to the practical, logistical and financial challenges of the proposed strategy, taking a health systems strengthening approach towards implementation.

Uganda faces a significant shortage of trained healthcare professionals, especially in the public sector and rural areas.^1^ As a result, the Ministry of Health (MoH) have supported delivery of the Village Health Team (VHT) model since 2001.^2^ VHTs are lay people, working in a voluntary capacity, acting as a link between the formal health sector and their communities.^3^ They are given basic training on major health issues, including childhood diarrhoea, malaria and pneumonia, and play a role in disease surveillance through activities such as data collection and reporting.^3^

Although the exact selection process for those wishing to become a VHT member varies depending on location, individuals commonly undergo selection starting in their own communities. After a period of sensitisation and consensus building among local stakeholders, a popular vote is held. To be selected as a VHT member, an individual must meet several criteria. He or she must be ‘above 18 years of age, a village resident, able to read and write in the local language, a good community mobiliser and communicator, a dependable and trustworthy person, someone interested in health and development and someone willing to work for the community’.^4 5^ Unlike formally trained healthcare professionals, such as doctors and nurses who are based at health facilities, VHT members are based in the communities in which they live and serve. This means the roles they play and the expectations that community members have of them are likely to be different.

Yet, despite reported successes of VHTs in improving and promoting health at a community level, challenges remain regarding their motivation, remuneration, training and retention.^2 6^ To try and address these issues, the Ugandan MoH has announced the planned roll out of a Community Health Extension Worker (CHEW) programme.^7^Modelled on the Ethiopian community health strategy, CHEWs will be paid, full-time health workers, with an O-level standard of education, aged between 18 and 35 and fluent in both local language and English.^3^ The initial aim of the MoH is to train and deploy 15 000 CHEWs across 7500 parishes nationally by the end of 2020.^3^ VHTs who will not be absorbed into the CHEW system will remain in their communities and continue to voluntarily provide health services, supported by CHEWs.^3^ Given the impending implementation of the programme, this article outlines some of the challenges we anticipate will arise based on our extensive work with VHTs over the past decade.

First, it is important to anticipate the potential tensions that may occur. The strict CHEW selection criteria, including the upper age limit of 35, will rule out many members of the community who have previously worked as VHTs. This could lead to feelings of animosity between new CHEWs and existing VHTs, who may feel overlooked and neglected, resulting in strained relationships. Furthermore, with the introduction of a paid cadre of community-based health workers, questions have been raised regarding whether VHTs will continue to be willing to volunteer their time. A study by Mbugua and colleagues, found that discrepancies in pay between volunteer and salaried community health workers in Kenya resulted in poor levels of motivation and higher levels of attrition in the unpaid cadre.^8^ Those responsible for implementing the CHEW policy should therefore consider strategies that have been shown to increase community health worker performance and motivation, in order to ensure existing VHTs do not feel undervalued.^9 10^ This might include the provision of tangible incentives, such as equipment and supplies, but also ensuring VHTs ideas, interests and relationships are duly considered so that tensions between the two cadres are minimised. Whichever incentives are chosen, they must be responsive to the needs of VHTs.

Additionally, there is potential for tensions to arise between CHEWs and community members. In a study by Musinguzi and colleagues, it was noted that community members in rural Uganda were distrusting of paid health workers, since they were concerned they might be profiting from referrals to health centres.^11^ Working closely with community members so that they understand the role of CHEWs will therefore be important.

The second challenge lies in the practical and logistical implementation of the programme. In the Mukono District where we work, there are nine parishes in the Ntenjeru subcounty alone, with a total population of approximately 550 000 people.^12^ Given the MoH have proposed allocating two CHEWs per parish, covering this number of households between 18 CHEWs will be extremely difficult, especially since they will spend just 60% of their time in the community and the remaining 40% in health facilities.^3^ Despite initially proposing to dissolve the VHT programme entirely and replace it with the CHEW model, the Ugandan MoH have now stated that CHEWs will supervise VHTs who will remain active in the community.^13^ Utilising both cares of health workers would make sense, given the logistical challenges of covering such a large population over a vast area, however, as previously mentioned, consideration must be given to the power dynamics and resulting conflicts that might arise.

It is also important to note the different, but complimentary roles that CHEWs and VHTs might play. Compared with the selection criteria for CHEWs, which largely focuses on the pre-service level of education, the selection criteria for VHTs places greater emphasis on community engagement, communication, trust and respect. Additionally, unlike CHEWs, VHTs are specifically selected by their own communities, meaning they could continue to play important roles in community mobilisation and advocacy.

The third challenge lies in the financial costs and sustainability of the programme. Implementing the CHEW strategy will cost an estimated US$102 million over a 5-year period, representing approximately 10% of the MoHs budget at present.^7^ Since the Ugandan public health system is already underfunded,^14^ introducing this paid cadre of CHEWs may not be possible without the support of external donors or a restructuring of the budget.^15^ Furthermore, although this proposed investment into community health must be welcomed, appropriate long-term funding into the health system at every level should also be encouraged so that this intervention is not approached in a vertical manner, but rather contributes to a wider health systems strengthening approach.

Finally, CHEWs and VHTs cannot be regarded as a panacea to address the dire shortage of health professionals seen across all cadres. Continued investment into the recruitment and training of other cadres of health workers must occur simultaneously. Second, it is important to note that the complex and multifaceted challenges facing community level healthcare in Uganda extend beyond the recruitment, training and deployment of CHEWs. As such CHEWs should not be seen as a ‘silver-bullet’ solution, but rather as one piece of a complex, multifaceted puzzle, which requires concurrent strengthening of other key areas known to influence community health.^16^ Taking this holistic approach will help to ensure that strong foundations are in place to maximise the potential benefits of the CHEW strategy.

In conclusion, prior to roll out of the CHEW programme, greater attention must be given to the practical, logistical and financial challenges of the proposed strategy. If these issues can be addressed and the relationship between CHEWs and VHTs harmonised, this initiative could represent an exciting opportunity to improve the attention and support given to community-based healthcare in Uganda.
